# The secrets of success

**DOI:** 10.7554/eLife.64379

**Published:** 2020-12-02

**Authors:** Kayla M Komondor, Anne E Carlson

**Affiliations:** Department of Biological Sciences, University of PittsburghPittsburghUnited States

**Keywords:** fertilization, capacitation, sperm, molecular heterogeneity, CatSper, in situ imaging, Mouse

## Abstract

Imaging sperm as they travel through the female reproductive tract has revealed new details about fertilization at the molecular level.

**Related research article** Ded L, Hwang JY, Miki K, Shi HF, Chung JJ. 2020. 3D in situ imaging of the female reproductive tract reveals molecular signatures of fertilizing spermatozoa in mice. *eLife* **9**:e62043. doi: 10.7554/eLife.62043

In mammals, sperm cannot fertilize eggs unless they spend time in the female reproductive tract, where they undergo a series of processes that are collectively called ‘capacitation’. During these processes, sperm change their swimming pattern, their cytosol becomes more basic, and the lipids that make up their plasma membrane change substantially (Florman and Fissore, 2015). The final stage of capacitation is the acrosome reaction, which involves the release of enzymes from a compartment in the head of the sperm called the acrosome. It is thought that these enzymes break down the ‘coating’ surrounding the egg so that fertilization can take place (Florman and Storey, 1982). It is also known that sperm from mice that do not produce a sperm-specific ion channel called CatSper do not undergo capacitation, so they are infertile (Lishko and Mannowetz, 2018; Ren et al., 2001).

CatSper ion channels allow Ca^2+^ to cross the cell membrane. The channels line up in a stripe pattern that begins in the midpiece of the sperm and extends down sperm tail (Figure 1; Chung et al., 2017). The opening and closing of CatSper channels is controlled by diverse cellular signals including membrane voltage, pH and the level of free Ca^2+^ inside the cell (Lishko and Mannowetz, 2018). The channel is comprised of at least ten subunits, including four proteins that form the central pore through which Ca^2+^ travels. Nearly all of these subunits are required for the channel to localize in the membrane and for male fertility (Hwang et al., 2019). For the most part, capacitation and CatSper channels have been studied by performing in vitro experiments in which mouse sperm are exposed to conditions that simulate the female reproductive tract. Now, in eLife, Jean-Ju Chung and colleagues at Yale University, the Czech Academy of Sciences and Boston Children’s Hospital – including Lukas Ded as first author – report on the differences between in vitro and in vivo capacitation (Ded et al., 2020).

**Figure 1.**
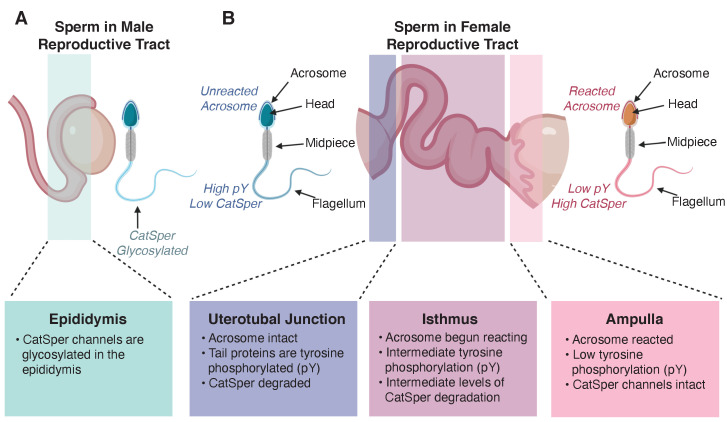
Changes in sperm during maturation and capacitation. (**A**) CatSper channels in the tail of sperm are glycosylated during maturation in the male epididymis. (**B**) Differences between sperm visualized in the female reproductive tract 8 hours post coitus. Sperm visualized near the uterotubal junction (the place where the uterus and the oviduct tubes meet, left) exhibited an intact acrosome, high levels of tyrosine phosphorylation (pY) in tail proteins, and degraded CatSper channels. Sperm in the isthmus (the part of the tube through which eggs pass from an ovary closest to the uterus, center) have already started their acrosome reaction, have intermediate levels of tyrosine phosphorylation in proteins in their tails, and intermediate levels of CatSper degradation. Finally, sperm in the ampulla (the part of the tube through which eggs pass closest to the ovary, where fertilization usually takes place) have reacted acrosomes, intact CatSper channels, and low levels of tyrosine phosphorylation in their tail proteins.

Figure created in BioRender.com.

To better understand how CatSper is regulated, Ded et al. checked whether proteins forming the channel were modified with either phosphoryl groups (phosphorylation) or carbohydrates (glycosylation). They found that the molecular weight of one of the CatSper pore-forming subunits, called CatSper1, increased as sperm matured in the epididymis (the highly convoluted tubes on top of the testes that lead to the duct through which sperm is expelled). When the protein was treated with deglycosylating enzymes, which remove carbohydrate modifications, the increase in molecular weight was reversed. This revealed that CatSper undergoes glycosylation (Figure 1A), which is thought to promote the migration and survival of sperm in the female reproductive tract (Ma et al., 2016).

Next, Ded et al. imaged mouse sperm that had been treated in vitro to induce capacitation and found that these sperm exhibited degradation of CatSper channels over time. This was unexpected because CatSper channels are required at the end of capacitation, close to fertilization. In sperm where CatSper was degraded, enzymes inside the sperm called serine/threonine proteases were cleaving the intracellular N-terminal domain of the CatSper1 subunit. This cleavage required high levels of Ca^2+^ inside the sperm cell which, in vivo, occur during capacitation.

Interestingly, this CatSper degradation was associated with the phosphorylation of tyrosine residues in proteins in the sperm tail: over 30 years ago it was proposed that tyrosine phosphorylation was an indicator of successful in vitro capacitation (reviewed in Florman and Fissore, 2015). By imaging sperm in vivo within the female reproductive tract, Ded et al. showed that the tails of sperm that had successfully traveled to the ampulla, the site of fertilization, were not tyrosine phosphorylated (Figure 1B). By contrast, sperm stuck in the uterus and unable to make the journey to the egg exhibited high levels of tyrosine phosphorylation. This supports recent evidence that tyrosine phosphorylation may not be required for capacitation (Alvau et al., 2016; Luño et al., 2013). Together these findings suggest that the tyrosine phosphorylation cascade that leads to CatSper degradation is a signature of sperm unable to fertilize.

Additional experiments imaging sperm within the female reproductive tract revealed other surprising findings. For example, sperm at the ampulla had already lost their acrosome, suggesting that sperm undergo the acrosome reaction prior to meeting the egg. By adapting the method they used to detect sperm (which involved making changes to a neural network that processed output from their imaging experiments) Ded et al. were able to assess the integrity of sperm as they navigated through the female reproductive tract. Sperm that successfully completed the journey to the egg in the ampulla had intact lines of CatSper extending over the length of their tail, whereas sperm undergoing CatSper degradation did not reach the site of fertilization.

Together these data show that CatSper degradation, the timing of the acrosome reaction, and tyrosine phosphorylation of proteins in the sperm tail differ substantially between in vitro and in vivo capacitation. This demonstrates that the conditions in the female reproductive tract are not well mimicked by current methods used to induce capacitation in vitro. Finding conditions that better mimic the natural environment of the sperm as it travels to the egg can improve in vitro capacitation. This can be used in the clinic to achieve greater success for people trying to get pregnant using assisted reproductive technologies.
